# Suitability of environmental indices in assessment of soil remediation with conventional and next generation washing agents

**DOI:** 10.1038/s41598-020-77312-7

**Published:** 2020-11-25

**Authors:** Barbara K. Klik, Zygmunt M. Gusiatin, Dorota Kulikowska

**Affiliations:** Department of Environmental Biotechnology, Faculty of Geoengineering, University of Warmia and Mazury in Olsztyn, 10-719 Olsztyn, Poland

**Keywords:** Environmental monitoring, Pollution remediation

## Abstract

Remediation of soils contaminated with metal must ensure high efficiency of metals removal, reduce bioavailability of residual metals and decrease ecological risk. Thus, for comprehensive environmental soil quality assessment, different indices must be used. In this study, suitability of 8 indices was used for soil highly contaminated with Cu (7874.5 mg kg^−1^), moderately with Pb (1414.3 mg kg^−1^) and low with Zn (566.1 mg kg^−1^), washed in batch and dynamic conditions with both conventional and next-generation washing agents. The following indices were used: modified contamination factor (*mC*_*f*_), modified contamination factor degree (*mC*_*deg*_), mobility factor (*MF*), reduced partition index (*IR*), potential ecological risk factor (*E*_*r,Z*_), modified potential ecological risk factor (*E*_*r,m*_), potential ecological risk index (*RI*_*Z*_) and modified ecological risk index (*RI*_*m*_). For m*C*_*f*,_ *mC*_*deg*_ and *IR* own classification scale was proposed. It was proven that most useful indices for assessment of soil pollution with metals were *mC*_*f*_ and *mC*_*deg*_. The *mC*_*f *_together with the *IR* allow to simultaneous assessment of soil pollution and stability for individual metals. These indices were appropriate for soil contaminated with different concentrations of metals, washed under both hydrodynamic conditions using various washing agents and different effectiveness of metals removal. Thus, they may be considered as most useful for evaluation of remediation method, feasibility of washing agent and assessing soil quality after washing.

## Introduction

An important sources of anthropogenic metals emissions are mining and metallurgy, which cause a severe environmental contamination in their immediate vicinity. Among the various elements of the environment, soils are those in which the effects of metals contamination persist for the longest time and remains even after removing the pollution source. Soil contamination with metals affects individual organisms and entire ecosystems, which is why they should be removed.

Among the available methods allowing the removal of metals there are soil washing (both in batch and dynamic conditions), with water or with washing agent (WA) of a high affinity for contaminant removal. Soil washing can remove contaminants adsorbed on soil particles by reducing the strength of bonding between pollutants and soil, increasing the hydraulic conductivity of the soil and the availability of pollution^1^. The decrease in capillary forces that occurs during the washing increases the mobility of the pollutants and the rate of their movement^2^.

Soil washing is well-established technology in many countries to remove permanently a variety of pollutants from highly contaminated soils^3^. The broad application of soil washing results from the possibility of using different washing agents dedicated to removal a specific pollutant and the ease in controlling the operational conditions. During soil washing, the contaminated soil is vigorously mixed with washing solution, which in turn reduces the remediation time and provides high pollutant removal. Another advantage of soil washing includes reducing the volume of soil to be treated by concentrating pollutants into fine particles, which reduces the remediation cost. Among different remediation methods, soil washing characterizes with a high public acceptance^4^.

This method requires soil excavation and soil transportation to a place of disposal. In practice, in situ methods, i.e. carried out directly in contaminated sites are also preferred^5^. For effective washing technology it is recommended to determine firstly the optimal parameters in the laboratory studies. This approach reduces the risk of failure and gives the opportunity to view and to resolve potential difficulties that may appear in the full-scale operation^6^. Such tests are conducted under batch and/or dynamic conditions (column leaching mode). In these methods, however, different mechanisms of metals transport occur^7^. In batch experiments, sorption/desorption of metals is determined under equilibrium conditions, whereas in column washing under non-equilibrium conditions, which run slower than under batch washing^8^. The efficiency of metals removal via soil washing in batch and dynamic conditions can vary widely. The efficiency of washing at the level of 70–80% is considered to be substantial. However, the efficiency above 50%, is still considered as cost-effective, and possible to use on a pilot and technical scale^6^.

During the past decades, studies have evaluated the effectiveness of removal of metals from the soil with various WAs, such as acids, salts and chelating agents^9–13^. Natural and environmentally friendly WAs, such as dissolved organic matter or soluble humic substances, can be also used to remove metals from contaminated soils with high efficiency^14–18^. Despite the process efficiency, some metals still remain in soil after treatment. Therefore, the quality of treated soil must consider several aspects, i.e. pollution level, metal stability and environmental risk. The key to assess soil contamination with residual metals can be using soil pollution indices. They are regarded as a tool and guide for an extensive evaluation of the state of the soil environment^19^. The indices are widely used and can be developed on different criteria, e.g. individual metals contamination (e.g. enrichment factor, EF; geoaccumulation index, I_geo_; contamination factor, *C*_f_)^19–22^ and multi-metals contamination (e.g. contamination degree, *C*_*deg*_)^21,23^. Other indices are based on the concentration of metals in their individual chemical forms, along with having great importance in determining the mobility of metals (as mobility factor, *MF*) and strength of metal binding with mineral-organic soil components (e.g. reduced partition index, *IR*)^24,25^.

Historically, most of these indices were used mainly for assessment of pollution level in sediments, then were adopted to soils contaminated with different metals and of different level of contamination^19,20,26^. There are some attempts to use them for assessment soil quality after remediation, but only with the use of selected, single indices, mainly concerning environmental risk^27,28^. However, as these indices were originally used for sediments, they are based on comparison between current metal concentrations with their geochemical background levels. Although, some of these indices can be used for urban and agricultural soils with elevated metal concentrations^19^, their using for highly contaminated soils can be inconclusive. For industrial objects connected with metal smelting, however, estimation of soil quality based on pollution indices is missing^26^.

In highly contaminated soils, despite a considerable decreasing of metal concentration after soil washing, the difference between residual metal concentrations in soil and geochemical background levels can still be high, indicating high pollution level, the same as for soil before remediation. However, taking into account that the purpose of remediation is decrease metal concentration to legislative level, regarding the current and, if possible, planned use of the land, more reliable for soil pollution assessment after remediation can be using the indices, at which current metal concentration will be referred to permissible levels in remediated soil instead of geochemical background. This was done in this study: modification of existing indices, based on soil pollution with single- and multi-metals, as well as own classification of the indices was proposed. Moreover, suitability of these modified indices, as well as indices based on strength of metal binding in soil and on ecological risk of metals, were assessed for soil after remediation. In order to check the indices at various levels of metal contamination and different remediation efficiency, all indices were calculated for: (1) soil of different degree of contamination by various metals (heavily polluted with Cu, moderately with Pb and weakly with Zn), (2) washed in batch and dynamic conditions, and (3) using both conventional (Na_2_EDTA) and next-generation WAs (dissolved organic matter, DOM; soluble humic-like substances, HLS and soluble humic substances, SHS).

## Materials and methods

### Soil

Soil taken from the top layer of agriculture area was used for the study. After air drying, the samples were ground and sieved through a 2 mm sieve. To obtain metals contamination similar to soils from the metallurgical area, the soil was spiked with a mixture of Cu(NO_3_)_2_, Pb(NO_3_)_2_ and Zn(NO_3_)_2_, according to the procedure of Chaiyaraksa and Sriwiriyanuphap^29^ and incubated for three months at room temperature. Metals values were assumed according to concentrations in soils around the Legnica smelter in Poland^18^. Because the aim of this study was to check the suitability of different indices to assess degree of soil contamination (and subsequently soil remediation), it was assumed that soil must be contaminated with metals of different concentration: from very high (several times above permissible concentration) to low (below permissible concentration). Such concentration range is necessary to select universal indices and to propose own classification scale for these indices. So, in this study soil was very highly contaminated with Cu, moderately with Pb and weakly with Zn.

Before soil treatment, soil properties were determined (Table 1). Based on the granulometric composition, soil was characterized as sandy loam with a low content of organic matter (3.4%) and slightly acidic pH (6.4). Total concentration of metals and their distribution were also shown in Table 1.

**Table 1:** Selected physicochemical soil properties (mean value ± standard deviation, n = 3).

**Soil properties**	**Value**		
Bulk density (g ml^−1^)	1.23 (± 0.08)		
Porosity (%)	54 (± 2.3)		
Sand (%)	56 (± 1.6)		
Silt (%)	39 (± 0.16)		
Clay (%)	5 (± 0.21)		
Texture	Sandy loam		
pH	6.4 (± 0.1)		
Organic matter (%)	3.4 (± 0.08)		
Cation exchange capacity cmol (+) kg^−1^	17.2 (± 0.7)		
Total Cu (mg kg^−1^)	7874.5 (± 23.06)		
Total Pb (mg kg^−1^)	1414.3 (± 11.6)		
Total Zn (mg kg^−1^)	566.1 (± 4.2)		
**Heavy metals distribution**	Cu (%)	Pb (%)	Zn (%)
Exchangeable and acid-soluble fraction (F1)	86	74	76
Reducible fraction (F2)	9	15	7
Oxidizable fraction (F3)	1	6	3
Residual fraction (F4)	4	5	14

### Washing agents (WAs)

Two groups of WAs were used in this study: (1) waste-derived in form of soluble organics from municipal sewage sludge (SS_WAs) and (2) commercially available Na_2_EDTA.

As SS_WAs, three different washing solutions were used: DOM, HLS and SHS. The previous study presented a detailed extraction procedure and characteristics of the used washing solutions^18,30^. Briefly, WAs were extracted (1:10 ratio (w/v)) with water (DOM) and 0.1 M NaOH (HLS and SHS) from the dried sewage sludge (105 °C). In addition, before the extraction of SHS, dissolved substances (i.e. sugars and proteins), waxes, and bitumen were removed from the sludge, as described in paper by Klik et al.^30^.

The methods of soil remediation and WAs were selected in such way to indicate various efficiency of metals removal from soil, e.g. Na_2_EDTA and HLS was extremely effective in removal of all metals, DOM was extremely effective in Cu removal and with low efficiency in Pb removal, SHS was effective in Pb removal etc.). Such scope of experiments allows to select the most universal indices of environmental quality of soil and to propose own scale for classification of these indices.

### Batch washing experiments

**Figure 1:**
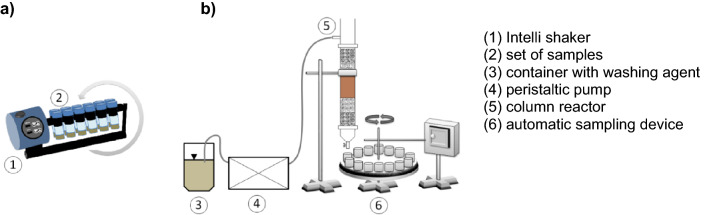
Set-up for metals removal from soil under (**a**) batch and (**b**) dynamic conditions.

The washing experiments under batch conditions were conducted in polyethylene tubes (50 mL) with soil to solution ratio of 1/40 (w/v). The samples were shaken in a mechanical shaker at 150 rpm at room temperature (Fig. 1a). The concentration of SS_WAs was 5 g C L^−1^. The concentration of Na_2_EDTA was typical used for soil treatment with the chelator (0.005 N; 0.64 g C L^−1^)^31^. In all WAs, the pH was adjusted to 4 and the contact time was 2 h (established in the previous study based on preliminary tests). After washing, samples were collected, centrifuged, and filtered through Whatman filter papers 42. The supernatants were acidified with HNO_3_ and kept at 4 °C before Cu, Pb, and Zn analysis.

### Dynamic washing experiments

The experiments were carried out in a column reactor equipped with a set of two sieves (supporting and closing) and two layers of rinsed gravel (2–4 mm and 1–2 mm) (Fig. 1b). The column reactor with length of 30 cm and radius of 1.5 cm was packed with 200 g gravel and 50 g of contaminated soil.

The tests were carried out at concentration and pH of WAs the same as in batch washing. Washings solutions were added to the reactor from the top and the eluate was collected from the bottom of the reactor by the automatic sampling device over time. Samples were taken every hour. Before proper soil washing procedure, distilled water was applied for 1 h to saturate the soil. Pore volume for the column reactor was 56.8 ml. The research was conducted at 2 different flow rates (0.5 ml min^−1^ and 1 ml min^−1^), for each washing solution for 24 h. All column experiments were conducted at room temperature, under laboratory conditions.

### The environmental indices for soil remediation assessment

In the present study, different environmental indices were used to assess the efficiency of batch and dynamic soil washing with SS_WAs agents and Na_2_EDTA. These indices, including total metals concentrations in soil and metals distribution in chemical fractions, were divided into three groups:

#### Indices based on soil pollution with metals

- single-metal soil pollution.

##### Modified contamination factor (m*C*_*f*_)

The contamination factor (*C*_*f*_), originally proposed by Hakanson^21^ for sediments, now is commonly used to assess soil contamination with metals^23,32–35^. For *C*_*f*_ calculation, mostly preindustrial or geochemical background concentrations of metals are used. The use of such reference values can be not reliable for assessment of metal pollution in highly contaminated before and after remediation. This is because in remediated soil achieving metal concentration on the level of geochemical background is impossible and it is not legally required. According to Polish Environmental Protection Law^36^, remediation is based on the removal or reduction the amount of substances causing the risk from soil and groundwater to the level of limit values, their control and limitation of migration, so that the contaminated area ceases to pose a threat to human health or the state of the environment, taking into account the current and the planned land use. Therefore, in this study for the assessment of metal pollution in contaminated and post-remediated soil, original *C*_*f*_ factor was modified to m*C*_*f*_, taking as reference of metal concentration, not preindustrial/geochemical background, but the limit values for metals in industrial soil given in a national regulation (here the Ordinance of the Minister of Environment^37^). The m*C*_*f*_ was calculated according to the following formula:

1 $$mC_{f}^{i} = \frac{{C_{s}^{i} }}{{C_{r}^{i} }}$$

where: C_s_ is the concentration of metals in soil sample, C_*r*_ is the limit value for a given metal in soil according to OME^37^: 600 mg kg^−1^ for Cu and Pb and 2000 mg kg^−1^ for Zn. The m*C*_*f*_ was categorized into five levels: m*C*_*f*_ $\le$ 1 means that soil meets the national quality standard for industrial sites, 1 $<$ m*C*_*f*_ $\le$ 2 means weakly to moderately contaminated soil, 2 m $<$ *C*_*f*_  $\le$ 3 means moderately to strongly contaminated soil, 3 $<$ m*C*_*f*_ $\le$ 4 means strongly to extremely contaminated soil and m*C*_*f*_ > 4 means extremely contaminated soil.

(b) multi-metal soil pollution.

##### Modified contamination degree (m*C*_*deg*_)

The overall assessment of sediment contamination with metals was introduced by Hakanson^21^ as a contamination degree (*C*_*deg*_). The *C*_*deg*_ is a sum of the *C*_*f*_ for individual metals. Like the *C*_*f*_, the *C*_*deg*_ was also used to assess soil contamination with metals^23,32–35^. Because in the present study, the *C*_*f*_ was changed for m*C*_*f*_, consequently the *C*_*deg*_ was changed for m*C*_*deg*_ and the m*C*_*deg*_ equation was modified as follows:

2 $$mC_{deg} = \mathop \sum \limits_{i = l}^{n} mC_{f}^{i} \times w$$

where: m*C*_*f*_ is the modified contamination factor calculated in Eq. (1), *n* is the number of metals in soil, *w* is factor weight resulting from the value of m*C*_*f*_, i.e. when m*C*_*f*_ ≤ 1 then *w* = 1 or when m*C*_*f*_ > 1 then *w* = *n*. The classification of m*C*_*deg*_ was simplified and two categories of soil quality were proposed: m*C*_*deg*_ ≤ *n* means that soil meets the national quality standards for industrial sites and m*C*_*deg*_ > *n* means that soil does not meet the national quality standards.

#### Indices based on the strength of metal binding in soil

##### Mobility factor (*MF*)

The *MF* is used to assess changes in metal mobility, and is defined as the ratio of metal concentration in the mobile fraction to the sum of metal concentrations in all analyzed fractions^25^. The *MF* (based on the BCR fractionation procedure) is expressed by the following equation:

3 $$MF = \frac{{C_{F1} }}{{C_{F1} + C_{F2} + C_{F3} + C_{F4} }} \times 100\%$$

where: C_*F1*,_ C_*F2*,_ C_*F3*,_ and C_*F4*_ are the concentrations of metal in exchangeable/acid soluble, reducible, oxidizable and residual fractions, respectively. With the *MF*, metal can be immobile (< 1%), weakly mobile (1–10%), moderately mobile (11–30%), highly mobile (31–50%) and very highly mobile (> 50%).

##### Reduced partition index (*IR*)

The *IR* informs about the strength of metals binding with mineral-organic soil components. When *IR* is close to 0, metal occurs in an easily soluble and exchangeable form, while *IR* close to 1 means that a given metal dominates in stable forms, mainly in the residual form^24,25^.

4 $$IR = \frac{{\mathop \sum \nolimits_{i = 1}^{k} i^{2} F_{i} }}{{k^{2} }}$$

where: *i* is the index number of the sequential extraction step, for the weakest to the strongest (*k*) (in the BCR procedure, *k* = 4), and *F*_i_ is the percentage content of the considered metal in a i-fraction. Because the *IR* corresponds with the *MF*, the following classification was proposed: lack of stability (*IR* ≤ 0.1), low stability (0.1 < *IR* ≤ 0.3), medium stability (0.3 < *IR* ≤ 0.5), elevated stability (0.5 <* IR* ≤ 0.7), high stability (0.7 < *IR* ≤ 0.9) and very high stability (*IR* > 0.9).

#### Indices based on ecological risk from metals

##### Potential ecological risk factor (*E*_*r*_)

The *E*_*r*_ assesses the impact of individual metal including their various toxicological effects^21^. The *E*_*r*_ is also used to evaluate the ecological risk for sediments and soils^38,39^. In the present study, the *E*_*r *_was calculated and compared according to the formula given by Hakanson^21^ and used in the study by Zhu et al.^40^ (in this study noted as *E*^*i*^_*r*,Z_) and according to our modification (in this study noted as *E*^*i*^_*r*,m_) using m*C*_*f*_ instead of *C*_*f*_:

5 $$E_{r,Z}^{i} = T_{r}^{i} \times C_{f}^{i}$$

6 $$E_{r,m}^{i} = T_{r}^{i} \times mC_{f}^{i}$$

where: *T*_*r*_ is the toxic-response factor for a given metal (5 for Cu and Pb, 1 for Zn, Hakanson^21^), m*C*^*i*^_*f*_ is the modified contamination factor.

Because in this study, similarly as in study by Zhu et al.^40^, the metals with the highest *T*_r_ (Hg, Cd, As) were not analyzed, we assumed the same classification of soil ecological risk as Zhu et al.^40^: low risk (*E*_*r*_ $\le$ 15), moderate risk (15 $<$ *E*_*r*_ $\le$ 30), considerable risk (30 $<$ *E*_*r*_ $\le$ 60), high risk (60 $<$ *E*_*r*_ $\le$ 120), and very high risk (*E*_*r*_ $>$ 120).

##### Potential ecological risk index (*RI*)

The *RI* is defined as the sum of *E*_*r*_ (Hakanson^21^). Because in the present study *E*^i^_*r*,Z_ and *E*^i^_*r*,m_ were established, therefore two *RI* were also calculated according to the Eqs. (7, 8):

7 $$RI_{Z} = \mathop \sum \limits_{i = l}^{n} E_{r,Z}^{i}$$

8 $$RI_{m} = \mathop \sum \limits_{i = l}^{n} E_{r,m}^{i}$$

According to the classification of the *E*_*r*,_ the classification of *RI* was different than that originally given by Hakanson^21^: low risk (*RI* $\le$ 50), moderate risk (50 $<$ *RI* $\le$ 100), considerable risk (100 $<$ *RI* $\le$ 200), and very high risk (*RI* $>$ 200)^41^.

### Analytical methods

The equilibrium pH of soil was measured with a pH meter (Hanna Instruments) in distilled water (1:2.5 ratio, m/V). The soil texture was determined using a Mastersizer 2000 particle size analyzer. The content of organic matter was determined by the Tiurin method and CEC was measured according to the Kappen’s method^42^. The concentration of metals in soil was determined with Flame Atomic Absorption Spectrometry (FAAS) using a spectrometer equipped with an automatic sample introduction and dilution system (SIPS). The samples were mineralized in a mixture of concentrated HCl and HNO_3_ before determination of total metals content. Mineralization was carried out in a microwave oven (MarsXpress, CEM). Metals distribution was determined according to a modified BCR procedure given by Pueyo et al.^43^, the following fractions were determined: exchangeable and acid-soluble fraction (F1); reducible fraction (F2); oxidizable fraction (F3); and a residual fraction (F4).

The obtained results are averages of three replicate measurements. The statistical analyses were performed with Statistica 13.1 (StatSoft, Inc.). One-way analysis of variance (ANOVA), followed by Tukey’s HSD test was employed to indicate significant differences between means for metal removal efficiencies and environmental indices in soil treated with different WAs under batch and dynamic soil washing. Validation of the data was carried out through comparing total metal content obtained by total digestion with sequential extraction of metals. The metal recovery ratio (R) was calculated as follows:

9 $${\text{R}} = \frac{{C_{{F1}} + C_{{F2}} + C_{{F3}} + C_{{F4}} }}{{C_{{total}} }} \times 100$$

where: *C*_*F1,*_* C*_*F2,*_* C*_*F3*,_ and *C*_*F4*_ is the concentration of metal in a given fraction, *C*_*total*_ is the total metal concentration.

## Results and discussion

### The removal efficiency of metals

Comparing the metals removal under different soil washing conditions, the batch washing was more efficient compared to dynamic washing. This may be due to the way of contact between the soil and WAs, which is more vigorous under batch conditions and a higher ratio of soil weight to volume of washing solution in comparison to dynamic washing. Although higher metal removal under batch than dynamic soil washing is typical phenomenon^44–46^, our intention was to show the different efficiency of metals removal and their different fractional distribution in batch- and dynamic-washed soil in terms of the possibility of assessing the usefulness of modified environmental indices for soils with different remediation efficiency.

In the present study, the soil was contaminated to the largest extent with Cu, which exceeded over 13 times permissible limit for industrial soils according to Polish legislative^37^. Therefore, Cu removal mostly affected the overall efficiency of soil remediation.

**Figure 2:**
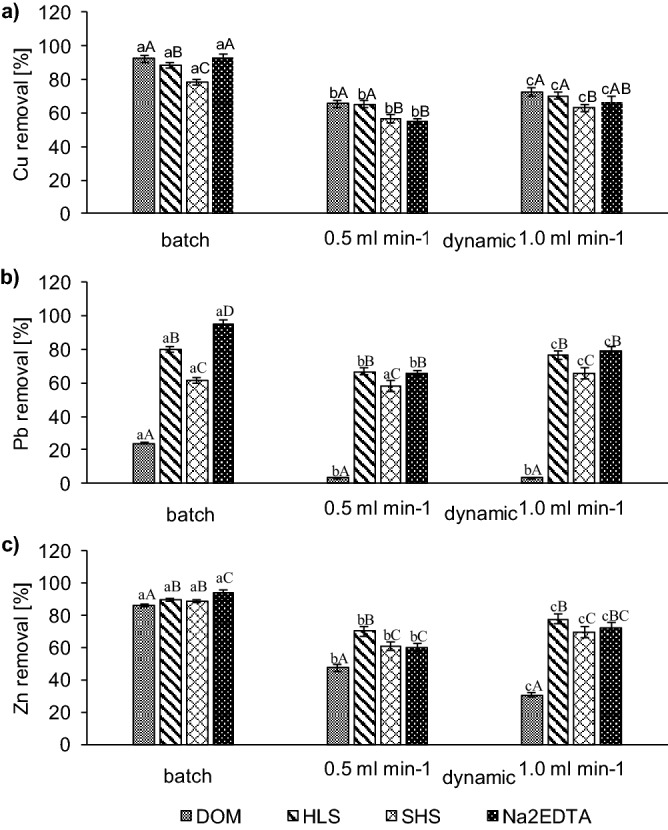
Comparison of the efficiency of metal removal from soil under batch and dynamic washing with DOM, HLS, SHS and Na_2_EDTA: (**a**) Cu, (**b**) Pb, and (**c**) Zn. Different letters indicate statistical differences at *p* < 0.05 (Statistica 13.1) between the efficiency of metal removal for different washing conditions (abc) and between the efficiency of metal removal with washing agents for a given washing condition (ABC).

Under batch conditions with all tested WAs, the average efficiency of Cu removal was 88% (ranging from 79% for SHS to 93% for Na_2_EDTA). Under dynamic conditions, the higher efficiency of Cu removal, and consequently lower residual Cu concentration in soil, was at 1 ml min^−1^ than at 0.5 ml min^−1^ flow rate (Fig. 2). DOM and Na_2_EDTA removed the highest amount of Cu from soil under batch conditions (> 92%), while under dynamic conditions, DOM and HLS were the most effective for Cu removal (the efficiency > 70%). Similarly, higher Pb removal was obtained in batch than dynamic conditions. However, in both hydrodynamic conditions, most effective in Pb removal were HLS and Na_2_EDTA. In contrast to Cu, DOM was able to remove only 24% of Pb under batch washing and only 3% (on average) under dynamic conditions. As in contaminated soil, Zn concentration was the lowest, compared to Cu and Pb, and its removal efficiency with DOM, HLS, SHS and Na_2_EDTA were high in both washing conditions. However, batch conditions were also more favourable than dynamic conditions. For example, the efficiency of Zn removal with HLS was 90% under batch conditions (in washed soil Zn remained mainly in F4 fraction) and 70–78% under dynamic condition at both flow rates.

The difference in metal removal with tested washing agents was related to their characteristics and affinity to individual metals. The washing agents extracted from sewage are composed of multiple organic compounds and functional groups. DOM contains mainly low-molecular-weight organics and fulvic acids. HLS contains both low-molecular-weight organics and macromolecular compounds (fulvic and humic acids), whereas SHS are mainly consist of fulvic and humic acids^3^ Among different organic functional groups, carboxyl (carboxyl-C) and amino (*N*-alkyl-C) groups readily interact with metals^47^. In all washing agents derived from sewage sludge, carboxyl (dissociated and undissociated) and unprotonated amino groups were the most abundant and the intensities in the infrared spectra for these groups decreased in the order DOM > HLS > SHS^30^. This can explain higher DOM efficiency in metal removal, especially Cu and Zn. In contrast, higher Pb removal with HLS and SHS than DOM suggests that its removal depends more on the presence of functional groups in macromolecular organic compounds in washing agents, especially humic acids than on functional groups in low-molecular-weight organics.

Because better removal of Cu, Pb and Zn under dynamic conditions was at higher flow rate, to compare the suitability of the many commonly used indices for assessment remediation efficiency and soil quality, the results from batch and dynamic washing at flow rate of 1.0 ml min^−1^ were selected.

### Patterns of metal distribution in soil washed under batch and dynamic conditions

Total content of metal measured in raw or treated soils is not a direct indicator of hazardous ecological consequences. Thus, each metal in the contaminated soil and soil after batch and dynamic washings was fractionated into different chemical fractions. According to Minkina et al.^48^, metals distribution should be determined in soil from each remediation action. The results on the changes in metals concentrations in individual fractions are shown in Table 2. The recovery values (R) for metals fractionation were in the ranges of 88.8–123.4% for Cu, 93.5–104.2% for Pb, and 92.6–105.1% for Zn. The values are considered to be satisfactory for the quality of the results on metals fractionation^49,50^.

**Table 2:** Total metal concentrations and their individual fractions in soil before and after soil washing under batch and dynamic conditions.

Metal	Washing agent	Washing mode	Total metal concentration (mg kg^−1^)	Metal fraction (mg kg^−1^)				R (%)
				**F1**	**F2**	**F3**	**F4**	
Cu*	Unwashed		7874.5	6655.2	660.4	96.0	327.6	88.8
	DOM	Batch	600.5	134.7	78.2	52.5	267.9	94.6
		Dynamic	2153.5	1236.3	391.4	90.8	311.6	94.3
	HLS	Batch	906.9	386.3	94.8	78.2	298.6	93.6
		Dynamic	2343.5	1486.3	412.5	84.9	306.4	97.7
	SHS	Batch	1685.1	1048.0	148.6	69.1	311.2	123.4
		Dynamic	2918.0	1968.0	461.3	107.5	317.6	97.6
	Na_2_EDTA	Batch	529.1	108.2	151.2	85.1	308.5	98.3
		Dynamic	2659.5	1808.2	451.2	89.1	320.1	99.9
Pb*	Unwashed		1414.3	1036.6	207.8	89.0	67.6	93.5
	DOM	Batch	1076.8	731.1	138.5	74.9	62.3	95.7
		Dynamic	1365.3	991.1	188.5	82.9	64.6	97.0
	HLS	Batch	281.3	84.2	43.6	79.3	62.1	93.7
		Dynamic	337.3	134.2	50.3	89.3	65	97.6
	SHS	Batch	545.9	290.6	63.1	80.9	64.7	104.2
		Dynamic	481.1	272.6	61.1	84.1	67	102.2
	Na_2_EDTA	Batch	71.5	3.9	3.2	24.8	42.7	99.1
		Dynamic	301.3	163.9	53.2	28.3	50.6	98.2
Zn*	Unwashed		566.1	423.7	40.6	17.8	77.3	101.4
	DOM	Batch	73.8	5.1	4.1	11.1	54.0	93.5
		Dynamic	393.1	251.2	36.1	16.4	70.6	95.2
	HLS	Batch	58.4	2.7	2.1	7.1	42.8	92.6
		Dynamic	127.1	22.1	22.1	15.1	68.8	100.7
	SHS	Batch	65.0	2.0	2.1	8.7	47.5	96.5
		Dynamic	171.8	61.9	30.6	16.7	67.2	102.6
	Na_2_EDTA	Batch	34.0	2.2	1.8	6.4	22.4	98.8
		Dynamic	158.2	86.8	23.7	13.4	42.4	105.1

*Limit values for industrial soils in Poland: Cu 600 mg kg^−1^, Pb 600 mg kg^−1^, Zn 2000 mg kg^−1^ (OME^37^), metal fractions: F1 is the exchangeable and acid soluble, F2 is the reducible, F3 is the oxidizable, F4 is the residual, R is the HM recovery ratio.

The results clearly demonstrated that the distribution patterns for individual metals depended on the soil washing conditions and the type of WA. The results have shown that the most mobile F1 fraction was considerably removed with the most of tested WAs under batch and dynamic conditions (Table 2).

The share of F1 fraction for Cu in soil washed under batch conditions was lower than under dynamic conditions, which is associated with lower process efficiency for these washing conditions (“The removal efficiency of metals” section). In batch conditions small share of Cu in F1 fraction was in soil washed with Na_2_EDTA (108.2 mg kg^−1^;16.6%) and DOM (134.7 mg kg^−1^; 25.3%), and the largest in soil washed with SHS (1048 mg kg^−1^; 66.5%). Under dynamic conditions, despite decreasing of total Cu, its distribution in all washed soils was similar to that in unwashed soil with the same order of fractions as F1 > F2 > F4 > F3 (Table 2). It must be emphasized that under dynamic conditions, the greatest decrease in the Cu concentration in the F1 fraction was recorded for DOM. It means that although in batch conditions effectiveness of DOM and Na_2_EDTA for Cu removal from F1 was similar, in dynamic conditions more favorable for Cu removal was DOM.

In the case of Pb, although batch washing was more effective than dynamic washing, distribution pattern of Pb in washed soil in both conditions was similar. However, this phenomenon was observed only with SS_WAs. A different pattern was noted in Na_2_EDTA-washed soil: in soil washed under batch conditions F4 fraction predominated, whereas in soil washed in dynamic conditions the F1 fraction of Pb predominated. The results indicate that batch soil washing was more favourable for Pb removal from individual fractions with all tested WAs.

The highest changes in fractional distribution were observed for Zn. Its distribution patterns in batch-washed soil with DOM, HLS, SHS and Na_2_EDTA were similar, with the largest share of Zn in F4 fraction (22.4–47.5 mg kg^−1^). In contrast, distribution patterns of Zn in dynamic-washed soil were more differential. Fraction F4, representing the lowest potential environmental risk, constituted the largest share in HLS-washed soil and in SHS-washed soil. However, the most mobile F1 fraction of Zn prevailed in DOM- and Na_2_EDTA-washed soil, followed by SHS- and HLS-washed soil (Table 2).

### Suitability of different environmental indices for assessment the quality of washed soil

#### Soil pollution with metals

**Figure 3:**
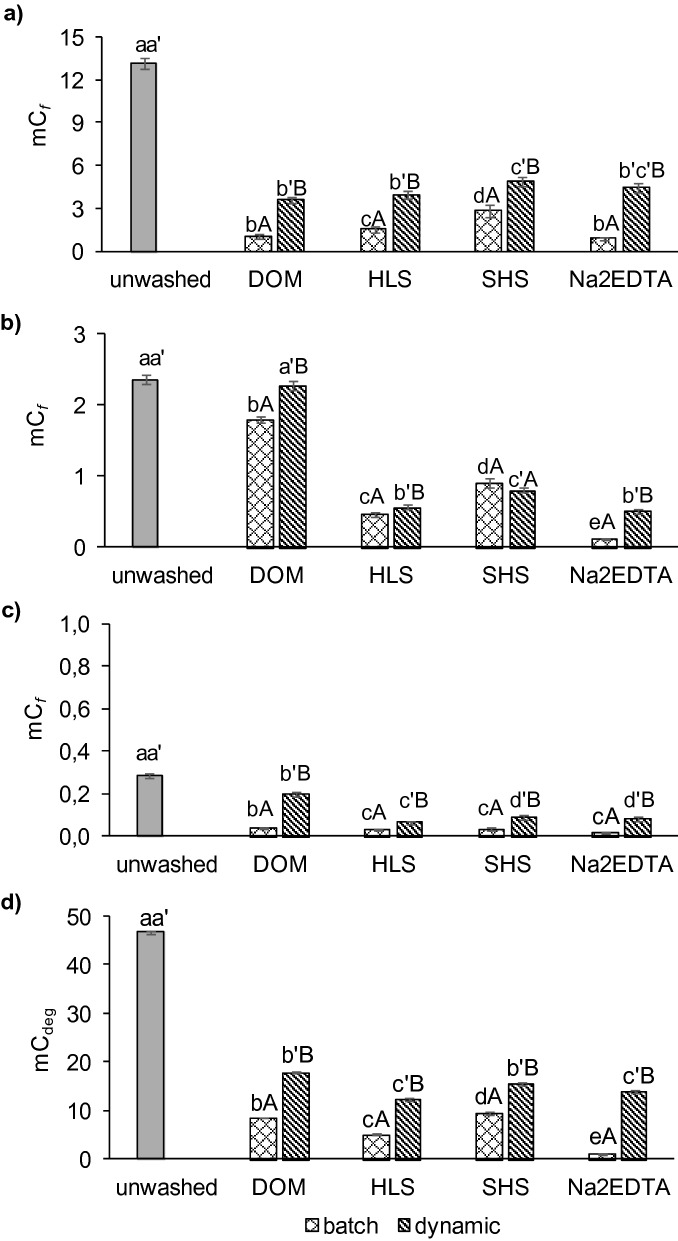
Assessment of the soil pollution with metals based on modified contamination factor (mC_*f*_ ) and modified contamination degree (m*C*_*deg*_): (**a**) mC_*f*_ for Cu, (**b**) mC_*f*_ for Pb, (**c**) mC_*f*_ for Zn, (**d**) m*C*_*deg*_. Significant differences (mean ± SD, p < 0.05, Statistica 13.1) for mC_*f*_ and m*C*_*deg*_ between soils washed under batch conditions (abc), between soils washed under dynamic conditions (a'b'c') and between washing conditions with the use of the same washing solution (AB) are marked with specific letters.

The soil pollution with metals before and after remediation was assessed with m*C*_*f*_ and m*C*_*deg*_ indices. With the m*C*_*f*_, unwashed soil characterized with different pollution levels for individual metals, from extreme contamination for Cu (m*C*_*f*_ = 13.1) (Fig. 3a) to moderate/strongly contaminated for Pb (m*C*_*f*_ = 2.4) (Fig. 3b). The soil has shown lack of contamination with Zn (m*C*_*f*_ = 0.28) (Fig. 3c).

Soil washing had a great effect on decreasing of m*C*_*f*_: higher decrease was observed in soil after batch than dynamic washing. In soil after batch washing with DOM and HLS, Cu showed weak to moderate contamination, however after washing with SHS soil was still highly contaminated with Cu (m*C*_*f*_ = 2.8). In soil washed under dynamic conditions with all WAs, the m*C*_*f*_ for Cu was above 3, reflecting strong to extreme contamination (Fig. 3a). For Pb, only soil washed with DOM under batch and dynamic conditions did not meet soil quality standards for industrial area (m*C*_*f*_ = 1.8 for batch washing and m*C*_*f*_ = 2.3 for dynamic washing) (Fig. 3c). After washing with other WAs, m*C*_*f*_ was < 1, meaning that soil met the national quality standard for industrial sites.

Assessing the soil contamination in relation to all metals (as m*C*_*deg*_), only soil washed with Na_2_EDTA under batch washing met the quality standards for industrial areas. In other soil samples, m*C*_*deg*_ was greater than 3 (from 5.0 to 18) (Fig. 3d). The results confirmed that total metal concentration is important for the efficiency of soil remediation and decreasing soil pollution level. Metal occurring at the highest concentration in soil before remediation (in this case Cu), affects mostly the m*C*_*deg*_ in remediated soil.

The necessity of modification of original pollution indices (*C*_*f*_ and *C*_*deg*_) given by Hakanson^21^ resulted from the fact that these indices were dedicated to sediments. Moreover, the classification of *C*_*f*_ and *C*_*deg*_ reflects the lower values of metal concentrations in contaminated sediments. Thus, the use of this classification for highly contaminated soils can be confused. For example, in this study Cu concentration in contaminated soil was 7874.5 mg kg^−1^ (13.1 times higher than OME^37^ limit), while Zn concentration was 566.1 mg kg^−1^ (3.5 times lower than OME^37^ limit). Calculated values of the original *C*_*f*_ for Cu and Zn were 787.4 and 6.4, respectively and in both cases, they indicated very high contamination (*C*_*f*_ ≥ 6). Another example is for soil washed in batch and dynamic conditions with Na_2_EDTA, for which the residual Cu concentration was 529.1 mg kg^−1^ (*C*_*f*_ = 52.9) and 2659.5 mg kg^−1^ (*C*_*f*_ = 265.9), respectively, indicating very high contamination (*C*_*f*_ ≥ 6) in soil washed in both conditions.

#### The strength of metal binding in soil

##### Mobility factor (*MF*)

**Figure 4:**
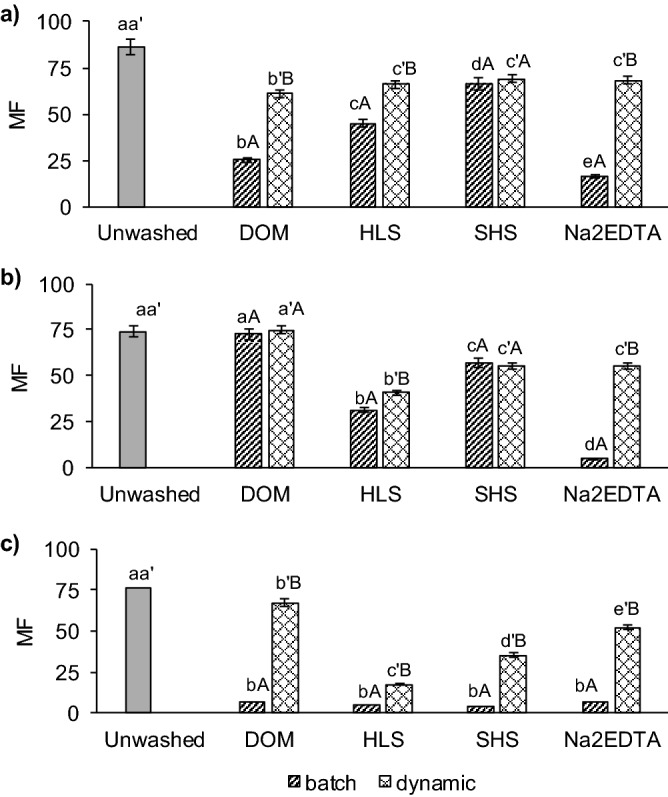
Mobility factor (*MF*) in unwashed soil and soil treated with different washing agents: (**a**) Cu, (**b**) Pb, and (**c**) Zn. Significant differences (mean ± SD, p < 0.05, Statistica 13.1) for *MF* between soils washed under batch conditions (abc), between soils washed under dynamic conditions (a'b'c') and between washing conditions with the use of the same washing solution (AB) are marked with specific letters.

In the present study, all metals in contaminated soil were highly mobile (74% < *MF* < 86%) (Fig. 4). Such superior values of the *MF* for Cu, Pb and Zn were associated with high share of metals in the exchangeable and acid soluble (F1) fraction. Metals in this fraction are bound to soil constituents with weak bonds and thus they are highly mobile^51^.

The values of *MF* have dropped significantly after washing, especially in batch-washed soil. The WAs had different ability to decrease the *MF* for individual metals. For Cu, the lowest *MFs* were in DOM-and Na_2_EDTA-washed soil (Fig. 4a). The low Cu mobility that has been observed for these agents corresponded to high metal stability in soil (as *IR*). In the case of HLS and SHS, the MFs were still high (45% and 66%, respectively). After soil washing under dynamic conditions, the *MFs* for Cu were higher for DOM, HLS and Na_2_EDTA than after washing under batch conditions, and they did not differ statistically (*MF* 67.7%, on average) (Fig. 4a). Comparable *MFs* for Cu were obtained in soil after washing with SHS under batch and dynamic conditions.

The highest effect on the *MF* decrease for Pb had HLS, SHS and Na_2_EDTA. It worth noticing that in soil washed with Na_2_EDTA under batch conditions, the *MF* for Pb was very low (5.2%), however, in soil washed under dynamic conditions it was 10 times higher (*MF* = 55.4%). In the case of HLS, the difference in *MFs* for both washing conditions was 9.5%, and Pb. For DOM and SHS, the conditions of soil washing did not affect the *MF* values of Pb. In soil washed with DOM, the *MF* of Pb was comparable as in unwashed soil (Fig. 4b).

The effect of washing conditions on the changes in *MF* was the greatest for Zn. With all WAs, the *MF* decreased from very high to low (5.4%, on average; p > 0.05) under batch soil washing. Despite decreasing the *MF* of Zn under dynamic conditions, the Zn mobility was still high for DOM-washed soil (*MF* = 67.1%) and Na_2_EDTA-washed soil (*MF* = 52.2%). The use of HLS and SHS led to decrease Zn mobility to low and medium level, respectively (Fig. 4c).

High values of the *MF* are typical for highly metal-contaminated soils. Kulikowska et al.^18^ demonstrated that in soil from metallurgical area in Poland, Cu and Zn showed very high mobility (*MF*), i.e. 73.1 and 70.6%, respectively. Cheng et al.^52^ found high *MFs* for Cu, Ni and Zn, i.e. 68%, 64% and 53%, respectively in soil from the electroplating factory (Dongguan City, China). The values of the *MF* for Cu, Ni and Zn were significantly decreased after soil batch washing with mixture of EDTA and oxalic acid to 22%, 25% and 23% for Cu, Ni and Zn, respectively. Decrease in metal mobility in soil after remediation is typical phenomenon. Gusiatin and Klimiuk^25^ demonstrated that significant decreasing of the *MF* values can be obtained through multiple batch soil washing. In soil spiked with Cu, Cd and Zn, the metals presented very high mobility (61% < *MF* < 84%), which was decreased below 10% for Cu and Cd and below 20% for Zn after triple soil washing with saponin biosurfactant. Also, Wang et al.^53^ showed that batch washing was very effective in decreasing Pb mobility with *N*,*N*-bis (carboxymethyl)-l-glutamic acid in mine soil, from 80 to 17%.

##### Reduced partition index (*IR*)

One of the tools to evaluate the stability of metals in soil is the *IR* index based on the metal distribution patterns^24,25^. Originally, the index was applied for assessment of metal stability in soil amended with different additives. Nowadays, it can be used also for soil remediated with soil washing^25^. An increase of the *IR* indicates metals redistribution in soil, from less to more stable fractions. In the case of BCR sequential scheme of metal fractionation, the F1 fraction is mobile, the F2 and F3 fractions are potentially mobile, and the F4 fraction is stable. Thus, the considerable increase of the *IR* is favoured by the increase of metal share in the most stable fraction.

**Figure 5:**
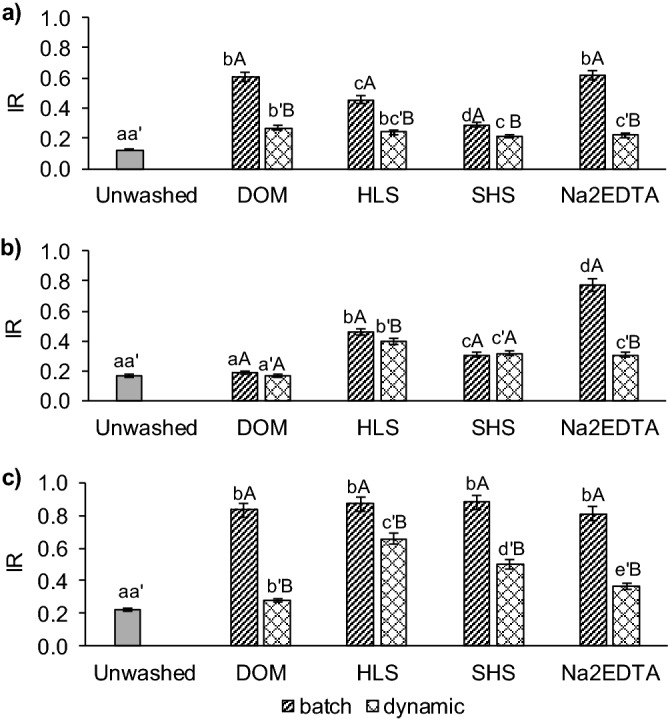
Assessment of the strength of metals bonding (as *IR*) in unwashed soil and soil treated with different washing agents: (**a**) Cu, (**b**) Pb, and (**c**) Zn. Significant differences (mean ± SD, p < 0.05, Statistica 13.1) for *IR* between soils washed under batch conditions (abc), between soils washed under dynamic conditions (a'b'c') and between washing conditions with the use of the same washing solution (AB) are marked with specific letters.

With the use of the *IR* index the changes in metal stability were captured. The values of the *IR* index for unwashed soil and soil washed with different WAs are shown in Fig. 5. The results indicate that *IR* values in unwashed soil were relatively low (*IR* = 0.12–0.22) indicating low stability of all metals. Soil washing increased the *IR* for all metals, except for Pb in DOM-washed soil (*IR* < 0.3). In general, metals in soil from batch washing showed higher bonding intensities compared to soil from dynamic washing. In additions, metals differed in bonding intensities in washed soil. The differences were strongly related to metal distribution patterns (“Patterns of metal distribution in soil washed under batch and dynamic conditions” section). Batch washing with DOM and Na_2_EDTA resulted in high stability of Cu (*IR* = 0.61 for both agents), whereas the stability of Cu in soil from dynamic washing was 2.6 times lower (*IR* = 0.26 for DOM and 0.22 for Na_2_EDTA). For Pb, the greatest differences in the metal stability between batch- and dynamic-washed soil (by *IR* = 0.47) was indicated for Na_2_EDTA. Medium Pb stability was in soil washed with SS_WAs under batch and dynamic conditions (*IR* = 0.5 and 0.4 for HLS and *IR* = 0.33 and 0.32 for SHS, respectively).

Batch soil washing was especially effective for Zn removal from mobile and potentially mobile fractions by all WAs, which resulted in high stability (*IR* > 0.8). In contrast, the Zn stability in soil after dynamic washing ranged from low for DOM (*IR* = 0.28) to elevated for HLS (*IR* = 0.66).

Studies evaluating the metal stability (based on *IR*) in soils after washing were conducted by Gusiatin et al.^54^. The authors have found that application of different biosurfactants as WAs (saponin, tannic acid, rhamnolipids) significantly increased the stability of Cd, Cu, Ni, Pb and Zn (based on the *IR*) in spiked soil. Similar observations were made by Cheng et al.^52^, who noticed an increase in the *IR* index in soils after washing with a mixture of EDTA and organic acids. The increase was associated with the effective removal of Cu, Ni, and Zn, mainly from F1 fraction, and increasing the share of the most stable fractions, which were removed less efficiently.

#### Ecological risk assessment of metals

In this study, calculated values of *E*_*r*_,_Z_ and *E*_*r*_,_m_ for individual metals, and the values of *RI*_Z_ and *RI*_m_ for overall risk assessment are shown in Table 3. Based on the values of the indices calculated for contaminated soil and soil washed under different hydrodynamic conditions with conventional and next-generation WAs, differing in the efficiency of soil remediation, it turned out that neither of these two indices is universal. The most visible discrepancy of these indices for remediated soil was observed for Cu. Despite a 13–15 fold decrease in the total Cu concentration in soil after washing with DOM and Na_2_EDTA, and the decrease in *E*_*r*_,_Z_ from 3937.3 to 300.3 and 264.6, respectively, the potential ecological risk was still very high (according to Zhu et al.^41^, *E*_*r*_,_Z_ ≥ 120 means very high potential ecological risk). Similar discrepancy was demonstrated for Cu removed under dynamic soil washing. For *E*_*r*_,_m_, its discrepancy was noted for Pb in contaminated soil and in soil after washing. In both soils, Pb has shown the same potential ecological risk. This means that both *E*_*r*_,_Z_ and *E*_*r*_,_m_ cannot be considered as universal for ecological risk assessment in soil contaminated with metals and soil remediated in batch and dynamic soil washing with SS_WAs and Na_2_EDTA under specific operational conditions.

**Table 3:**
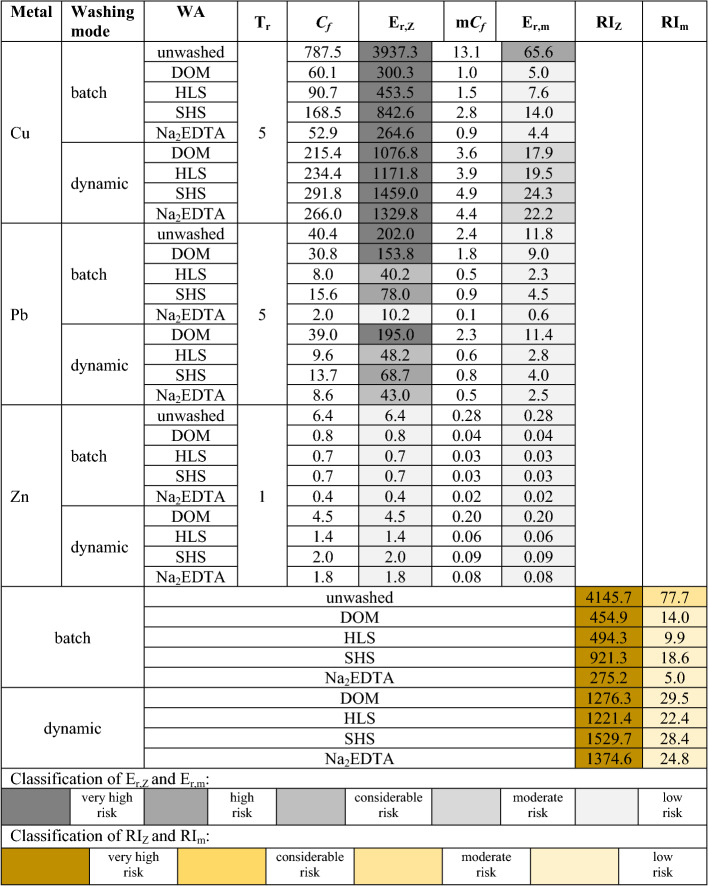
Comparison of potential ecological risk factors (*E*_*r*,Z_,* E*_*r*,m_) and potential ecological risk indices (*RI*_Z_, *RI*_m_) in soil before and after washing.

## Conclusions

Considering legislative purposes of soil remediation, in this study, the use of current metal concentration in relation to permissible levels in soil instead of geochemical background/pre-industrial level was proposed for calculation the indices of soil pollution after remediation. In addition, own classification scale for soil pollution indices and metal stability in soil was proposed. The *mC*_*f*_ and *mC*_*deg*_ were useful to identify soil pollution with metals. The mC_*f*_ together with the *IR* characterize individual metals in terms of their pollution and stability degree in soil. The indices clearly distinguished the environmental quality of soil after remediation under different hydrodynamic washing conditions with Na_2_EDTA and SS_WAs. Thus, they may be an useful tool for assessment of soil remediation with different WAs. Their usefulness requires further verification in case of treatment of other types of soils contaminated with different levels of metals and/or metalloids.
